# Mechanistic Insight into PVAT Browning as a Protective Factor in Thoracic Aortic Aneurysm

**DOI:** 10.1007/s11883-025-01373-0

**Published:** 2026-01-13

**Authors:** Wenjuan Mu, Zhenguo Wang, Y. Eugene Chen, Lin Chang

**Affiliations:** 1 Department of Internal Medicine, Cardiovascular Center, University of Michigan Medical Center, Ann Arbor, MI 48109 USA; 2 Department of Cardiac Surgery, Cardiovascular Center, University of Michigan Medical Center, Ann Arbor, MI 48109 USA

**Keywords:** TAA, PVAT plasticity, Dysfunction, Phenotypic swift, Remodeling

## Abstract

**Purpose of Review:**

This review aims to provide a comprehensive overview of emerging evidence supporting the protective effects of perivascular adipose tissue (PVAT) browning in the pathophysiology of thoracic aortic aneurysm (TAA).

**Recent Findings:**

PVAT is increasingly recognized as an active regulator of vascular homeostasis. During cardiovascular disease (CVD), PVAT undergoes a phenotypic shift from a protective brown/beige state to a dysfunctional white phenotype, contributing to vascular remodeling. Accumulating data supports beneficial effects of PVAT browning on key mechanisms involved in TAA development, including endothelial dysfunction, vascular smooth muscle cell phenotypic switching, adventitial remodeling and PVAT phenotypic shift. Studies highlight PRDM16 as a central regulator of PVAT browning, with its deficiency promoting PVAT dysfunction, adventitial fibrosis, and TAA formation.

**Summary:**

Strategies aimed at enhancing PVAT browning represent a promising therapeutic direction. However, significant gaps remain in our understanding of human PVAT biology, its interaction with the aortic wall, and the development of specific imaging tools or biomarkers. Further research is needed to clarify PVAT’s role in TAA pathophysiology and to advance browning-based interventions.

**Graphical Abstract:**

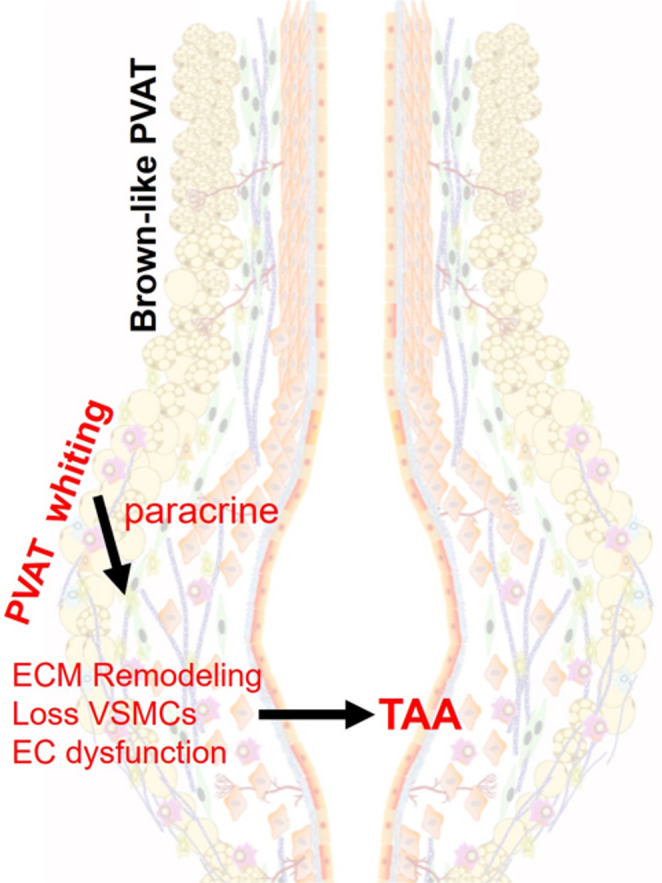

## Introduction

Thoracic aortic aneurysm (TAA) is a serious vascular disorder characterized by abnormal dilation of the thoracic aorta, which can lead to life-threatening complications such as dissection or rupture. Often referred to as a “silent killer,” it typically progresses without symptoms, leaving most patients unaware until sudden cardiovascular events occur. The incidence of TAA is estimated at 5.3 cases per 100,000 individuals annually, with a prevalence of 0.16% [1]. However, because it often goes undetected, especially in regions with limited access to advanced imaging, its true incidence and prevalence are likely significantly underestimated. General risk factors include male sex, hypertension, dyslipidemia, smoking, advanced age, and genetic variants [2, 3]. Due to the limited understanding of the underlying pathophysiology of TAA, there are currently no effective drug-based therapies. Preventive strategies primarily focus on managing cardiovascular risk factors, while definitive treatment relies on surgical interventions such as endovascular or open repair. However, the long-term prognosis following these procedures remains suboptimal. Overall, 5-year survival rates after either endovascular or open repair range from approximately 62% to 72% [4], with 10-year survival declining to around 50% in many patient cohorts [5, 6]. Therefore, a comprehensive understanding of the pathophysiology of TAA is essential for identifying potential pharmacological targets and improving the prognosis of patients who undergo surgical treatment. In this review, we systematically summarized recent research advances in TAA and highlighted novel mechanistic insights.

## The Cellular Landscape of TAA: Traditional and Emerging Contributors To Disease Progression

TAA can affect different segments of the aorta, including the aortic root, ascending aorta, aortic arch, and descending aorta, with underlying pathophysiological mechanisms varying by these locations. For example, ascending TAA are usually non-arteriosclerotic and strongly associated with genetic factors including connective tissue disorders and familial forms, and generally not related to traditional arterial risk factors. Conversely, descending TAA more commonly exhibit arteriosclerotic features, including irregular and calcified lesions of the aortic wall [7, 8] This distinction reflects differences in the structural and cellular composition of the aortic segments, as well as variations in the biochemical properties and embryological origins of vascular cells, all of which influence disease mechanisms [2, 9]. Nevertheless, TAA development at different anatomical sites typically results from a combination of risk factors that promote degeneration of the aortic wall, with distinct pathological changes observed depending on location.

The aortic wall is organized into three distinct layers: the intima, media, and adventitia. The intima comprises a single layer of endothelial cells (ECs) lining the lumen, which regulate vascular tone and barrier function. The media consists primarily of vascular smooth muscle cells (VSMCs), which are responsible for maintaining structural integrity and contractility. The adventitia, the outermost layer, is rich in fibroblasts that contribute to extracellular matrix (ECM) production and intercellular signaling. Historically, VSMCs have been considered the key cell type involved in the pathogenesis of TAA. Pathophysiological changes in VSMCs are viewed as central drivers of aneurysm formation and include several critical mechanisms:

### Phenotypic Switching

VSMCs transition from a contractile phenotype, characterized by the expression of contractile proteins, to a synthetic or secretory phenotype, characterized by increased proliferation, migration, and ECM-degrading activity [10, 11].

### Apoptosis and Senescence

Excessive VSMC apoptosis and cellular aging reduce cell numbers within the media, thereby weakening matrix support and promoting medial degeneration [10, 12].

### ECM Remodeling

Synthetic VSMCs secrete matrix metalloproteinases (MMPs), ADAM/ADAMTS proteases, and cathepsins that degrade key structural components such as collagen, elastin, and proteoglycans, ultimately compromising the integrity of the aortic wall [13–15].

### Inflammatory Activation

VSMCs produce pro-inflammatory cytokines and chemokines (e.g., IL-6, MCP-1, TNF-α), which recruit immune cells and amplify local inflammation [15, 16].

 Collectively, these processes disrupt the structural framework of the thoracic aorta, leading to aneurysm formation and increasing the risk of life-threatening complications. In addition to VSMC dysfunction, EC dysfunction also plays a pivotal role in TAA development. EC dysfunction is characterized by impaired barrier function, decreased nitric oxide (NO) bioavailability, increased oxidative stress, and inflammation, all of which contribute to progressive damage of the aortic wall [17–19]. Moreover, adventitial fibroblasts are increasingly recognized for their active involvement in TAA pathophysiology. These cells contribute to abnormal ECM turnover through excessive and disorganized collagen production, impaired cross-linking, and elevated MMP expression, further promoting aortic dilation and degeneration [20, 21].

While ECs, VSMCs, and fibroblasts are the primary structural and functional cell types in the aortic wall, recent research has increasingly recognized additional cell populations as important contributors to aortic remodeling during TAA development. For example, mesenchymal stem cells (MSCs) have multipotent capabilities and can differentiate into various cell types participating in regeneration, or pathological repair and remodeling in the aortic wall [22, 23]. Immune cells (including macrophages, T cells, neutrophils, etc.) infiltrate the aortic wall during aneurysm development and contribute to inflammation, matrix degradation, and remodeling processes [24, 25]. Pericytes, the mural cells that envelop the vasa vasorum, contribute critically to microvascular stability, angiogenesis and remodeling. Human TAA study shows reduced pericyte coverage of the vasa vasorum due to increased detachment [26]. Loss of pericytes is linked to vessel fragility, impaired barrier function, and fibrosis, contributing to TAA pathology [27, 28]. However, the nature of pericyte phenotypic changes during TAA remains debated. Huang et al. reported a shift toward a more contractile pericyte phenotype in ascending TAA and showed that FGF2 maintains a less contractile, pro-angiogenic state via MAPK and PI3K–AKT signaling [29]. In contrast, Mohammed et al. found that TAA pericytes were less contractile with increased proliferation, migration, suppressed angiogenesis, and MEK pathway overactivation. MEK inhibition with PD0325901 restored pericyte contractile and angiogenic properties and mitigated TAA progression [26]. Furthermore, functional studies revealed that pericytes from the human aortic vasa vasorum increase contractility on healthy ECM but not aneurysmal ECM, suggesting compromised contractile function in TAA [30].

In addition to these vascular cell types, increasing evidence indicates that metabolic factors, particularly free fatty acids (FFAs), also influence aneurysm pathology. Elevated total FFAs and saturated fatty acids have been associated with increased AAA risk [31], although individual FFAs appear to exert distinct effects. For example, a study of aortic tissue from Marfan syndrome (MFS) patients reported that increased oleic acid levels upregulate iNOS signaling, promoting endothelial dysfunction and aneurysm formation [32]. Palmitate has been shown to inhibit lysyl oxidase (LOX) maturation and activity, thereby weakening extracellular matrix integrity and contributing to AAA development [31]. In contrast, propionate exerts a protective effect by enhancing regulatory T-cell recirculation within the colonic lamina propria, which dampens inflammation and mitigates AAA progression [33].

Taken together, the interplay between diverse cellular contributors and metabolic signals forms a complex cellular environment that contributes to the structural and functional deterioration of the aorta, thereby promoting TAA development through multiple pathological mechanisms. Nevertheless, these insights have yet to be translated into effective pharmacological therapies for TAA. Therefore, it is necessary to expand the investigation into the mechanisms underlying the pathological processes of TAA to identify novel therapeutic targets. Recent studies highlight perivascular adipose tissue (PVAT) as a dynamic regulator of vascular health through its outside-in signaling, which influences vascular tone, remodeling, and inflammation. This emerging recognition reflects a shift in perspective, with the potential for PVAT to act not only as a contributor to TAA pathogenesis but also as a promising therapeutic target.

## Phenotypic Plasticity and Pathological Remodeling of PVAT in CVD

Adipose tissue broadly functions as an endocrine organ influencing multiple pathophysiological processes of cardiovascular disease (CVD) through systemic endocrine signaling [34]. Among the different fat depots, PVAT is unique due to its direct anatomical location surrounding the outer wall of most blood vessels, from the aorta to the dermal microvasculature [35]. Unlike subcutaneous or visceral fat, PVAT not only provides vital structural support to blood vessels but also acts as an active endocrine and paracrine organ. Its close physical integration with the aorta gives it a unique capacity to modulate vascular function and homeostasis through the local paracrine release of various bioactive molecules, including adipokines [36–38], cytokines [39, 40], and growth factors [41, 42], which collectively orchestrate vascular health and contribute to the pathophysiology of CVD.

PVAT exhibits significant phenotypic, genotypic, and functional heterogeneity depending on its anatomical location and varies across species [43, 44]. In rodents, PVAT surrounding the thoracic aorta closely resembles brown adipose tissue (BAT) [45], characterized by abundant mitochondria and thermogenic capacity. In contrast, human thoracic aortic PVAT more closely resembles beige adipose tissue (BeAT) [46], which retains BAT-like thermogenic properties but displays morphological features similar to white adipose tissue (WAT), such as larger lipid droplets. Importantly, PVAT demonstrates phenotypic plasticity, with the ability to transition between brown-like and white-like states in response to physiological or pathological stimuli. This plasticity influences PVAT’s role in vascular homeostasis and disease progression.

Although the precise pathological alterations of human PVAT during CVD remain largely undefined, emerging evidence from murine models suggests that promoting PVAT browning may have protective effects against CVD. Mouse models revealed that healthy PVAT resembles BAT or BeAT, characterized by high metabolic activity that facilitates lipid clearance and exerts paracrine control over vascular tone. In response to pathogenic stimuli or during the early stages of CVD, PVAT undergoes adaptive browning or beiging, a process that serves as a protective mechanism to counteract vascular remodeling [47], atherosclerosis [48] and hypertension [49]. However, as pathological stimuli persists and disease progresses, PVAT function deteriorates. Chronic inflammation and oxidative stress drive a phenotypic shift marked by diminished thermogenic capacity, hypertrophy of adipocytes, and alterations in the adipokine secretome—hallmarks of PVAT whitening [50–52]. In advanced CVD, PVAT may paradoxically exhibit increased expression of brown adipocyte markers and reduced adipocyte size [48]; yet this apparent “re-browning” does not reflect healthy metabolic activation. Instead, it signifies a compensatory response to pathological stress, characterized by increased adipocyte apoptosis and necrosis, along with extensive tissue remodeling. The persistent inflammatory milieu and metabolic disturbances induce myofibroblast accumulation and ECM deposition, resulting in PVAT fibrosis [53, 54]. Dysfunctional PVAT increasingly secretes pro-inflammatory cytokines, chemokines, and other mediators, thereby exacerbating vascular inflammation and accelerating the progression of CVD [55, 56].

Due to limited availability of human PVAT samples across different stages of CVD, our understanding of its pathogenesis in humans remains incomplete. Nonetheless, in alignment with observations from mouse models, accumulating clinical studies document a brown-to-white phenotypic shift in PVAT among patients with atherosclerosis [57], coronary artery disease [58], aortic valve disease [59], and TAA [60]. Moreover, the PVAT Fat Attenuation Index (FAI), a clinically validated imaging biomarker of coronary inflammation, appears to reflect the pathological “re-browning” described in murine studies. Specifically, FAI captures smaller, lipid-depleted adipocytes in PVAT adjacent to inflamed vessels, which is due to vascular inflammation-driven lipolysis and impaired adipogenesis [61, 62]. These parallels suggest that, despite interspecies differences, PVAT undergoes similar maladaptive remodeling in both humans and mice during CVD progression, highlighting the translational potential of findings from mouse models.

## The Therapeutic Promise of PVAT Browning

In contrast to the BAT-like characteristics of thoracic PVAT, PVAT in the abdominal aortic region (or abdominal PVAT) exhibits predominantly beige-like features in both morphology and gene expression profile [39, 44]. Despite its thermogenic capacity, the white-like characteristics of abdominal PVAT are thought to influence local vascular susceptibility to disease. For example, in both large animals and humans, the abdominal aorta is more prone to atherosclerosis than the thoracic aorta [63]. Transplantation of thoracic PVAT to abdominal aorta has been shown to mitigate aneurysm development [64]. Moreover, genetic ablation of healthy PVAT accelerates atherosclerotic lesion formation [45, 56]. These studies indicate that BAT-like characteristics may confer greater resistance to CVD development. Indeed, a growing number of studies have documented that PVAT browning inducers such as mild cold exposure, growth factors (e.g., BMP4 [57]), mitochondrial proteins (e.g., Rps3a [58] and mitoNEET [58]), β3-adrenergic receptor agonists (e.g., CL316243 [47]) and thermogenic regulators(e.g., PGC1a [65] and PRDM16 [47]) can promote PVAT browning. This process reduces vascular inflammation and helps prevent atherosclerosis, hypertension, and pathological vascular remodeling. The protective effects of brown-like PVAT signaling on aortic wall pathology suggest that PVAT browning could be a potential protective mechanism against TAA development, highlighting the need for further investigation.

Below, we provide a comprehensive overview of the outside-in signaling of PVAT and its effects on ECs, VSMCs, and fibroblasts within the adjacent aortic wall. Additionally, we discuss the PVAT phenotypic shift and how PVAT browning may modulate these cellular processes, thereby contributing to vascular homeostasis and the pathophysiology of TAA.

## The Role of PVAT in Regulating Endothelial function, Inflammation and Atherosclerosis Progression and Hypertension Development

Atherosclerosis is a significant contributing factor to the development and progression of many TAA, particularly in the descending thoracic aorta, where plaque buildup weakens the aortic wall over time [66]. This process promotes inflammation and MMP-mediated ECM degradation, which overlap with pathological mechanisms seen in TAA progression. Thus, preventing atherosclerosis can reduce the risk or slow the progression of TAA influenced by atherosclerotic risk factors. Additionally, hypertension is highly associated with TAA development and progression. Studies show that both diagnosed and occult central hypertension (high central aortic blood pressure) are prevalent in patients with TAA and correlate with larger aneurysm size and faster aneurysm growth. Central systolic blood pressure appears especially important, being independently associated with aneurysm expansion even when brachial blood pressure is normal [67]. This suggests that hypertension contributes causally to aneurysm progression by increasing mechanical stress on the aortic wall. Endothelial dysfunction caused by disturbed or low shear stress is a key early event in the initiation of atherosclerosis and hypertension. Browning PVAT has been shown to maintain the anticontractile (vascular relaxing) effect of PVAT, improve endothelial function, and reduce hypertension and atherosclerosis development in experimental models [36, 49, 57].

Nitric oxide (NO) plays a fundamental role in maintaining endothelial health. PVAT regulates NO bioavailability both directly and indirectly. Directly, PVAT expresses nitric oxide synthase (NOS) enzymes [68], and removal of PVAT has been shown to reduce basal NO production in small arteries from healthy individuals [69]. Indirectly, PVAT modulates endothelial function via paracrine signaling. For example, healthy PVAT secretes high levels of adiponectin, which enhances endothelial nitric oxide synthase (eNOS) phosphorylation and activity, thereby improving endothelial function, supporting blood pressure regulation, and attenuating atherosclerosis [70–72]. Conversely, obesity induces PVAT-derived WNT5a secretion, which promotes arterial oxidative stress, reduces NO bioavailability and worsens endothelial dysfunction [73]. Lactoferrin has been shown to ameliorate obesity-induced endothelial dysfunction and PVAT whitening by inhibiting Tak1-dependent interleukin-18 (IL-18) secretion from perivascular adipocytes, thereby relieving IL-18–mediated suppression of NO production and restoring endothelial function [74].

In addition to regulating NO bioavailability, PVAT plays an active role in modulating endothelial inflammation. Obesity promotes the accumulation of myeloperoxidase (MPO)-releasing myeloid cells within PVAT, and MPO levels correlate positively with body weight and endothelial dysfunction in both obese patients and animal models. MPO deficiency, in turn, reduces the frequency of myeloid cells in PVAT, enhances PVAT browning and adiponectin secretion, and improves endothelial function while reducing arterial stiffness. In vitro, MPO-treated human adipocytes exhibit decreased expression of adiponectin and brown adipocyte markers, alongside increased inflammatory signaling [75]. Similarly, deficiency of G protein-coupled receptor kinase 2 (GRK2) in myeloid cells preserves endothelium-dependent relaxation induced by acetylcholine or insulin. Mechanistically, downregulation of GRK2 in myeloid cells attenuates high-fat diet (HFD)-induced infiltration of macrophages and T lymphocytes into PVAT and lowers the expression of pro-inflammatory mediators such as tumor necrosis factor-α (TNF-α) and NADPH oxidase 1 (Nox1), thereby mitigating endothelial dysfunction [76]. Furthermore, in our previous study using adipose tissue-specific and brown adipocyte-specific BMP4 knockout mice, we demonstrated that PVAT BMP4 deficiency leads to reduced expression of brown adipocyte marker genes, impairing lipid metabolism in perivascular adipocytes. This impairment promotes the secretion of pro-inflammatory factors by PVAT adipocytes (especially IL-1β), which in turn increases endothelial inflammation and accelerates the development of atherosclerosis [57]. We further demonstrated that BMP4-driven PVAT browning plays a protective role in hypertension by preserving endothelial function [77].

## PVAT Browning Prevents VSMC Phenotypic Switch and Vascular Remodeling

Vascular remodeling is often a maladaptive response to pathophysiological stimuli, defined by abnormal fibrosis and thickening of the aortic wall, which may theoretically increase the risk of TAA dissection and rupture. However, this remodeling process also involves ECM degradation and disorganization, resulting in compromised aortic wall integrity. A critical event in vascular remodeling is the VSMC phenotypic switch. In response to stimuli associated with TAA risk factors, such as injury, inflammation, or genetic predisposition, contractile VSMCs transition to a synthetic phenotype. These synthetic VSMCs exhibit increased secretion of MMPs, which degrade ECM components and contribute to weakening and structural degradation of the aortic wall.

Emerging evidence implicates PVAT as a critical regulator of this process, particularly under obese conditions. PVAT mass is markedly increased in obesity, and conditioned medium derived from the PVAT of obese rats has been shown to promote VSMC phenotypic switching [78]. This effect is likely mediated by bioactive molecules secreted from PVAT, as it produces growth factors such VEGF, TGF-β, and PDGF-BB—all of which are known to stimulate VSMC proliferation and migration [79–81]. PVAT-conditioned medium has been shown to upregulate VEGF receptor expression in VSMCs and enhance their proliferative capacity [80]. Leptin, abundantly secreted by PVAT in obesity, may be a key mediator of this phenotypic shift. The VSMC phenotypic switching induced by obese PVAT can be attenuated by pretreatment with a leptin receptor antagonist [78]. Furthermore, PVAT-derived leptin facilitates interleukin-18 binding to its receptors on VSMCs, promoting VSMC apoptosis and contributing to abdominal aortic aneurysm (AAA) formation [82]. Additionally, dysfunctional PVAT increases the release of factors such as visfatin [83], resistin [84], WNT5A [73], and extracellular vesicles (EVs) [85, 86] containing microRNAs which have also been implicated in promoting VSMC proliferation, migration, and phenotypic modulation. In contrast, FAM19A5, a factor negatively correlated with obesity, has been identified as a protective molecule. Adipocyte-derived FAM19A5 inhibits VSMC proliferation and migration, contributing to reduced neointima formation following vascular injury [87].

While dysfunctional PVAT exacerbates vascular pathology, browning of PVAT appears to offer vascular protection. Browning has been shown to reduce inflammation, restore vascular structure and function, and ultimately prevent vascular remodeling. In mouse models of endovascular injury, genetic silencing of PRDM16 inhibits PVAT beiging, resulting in heightened inflammation and vascular remodeling. In contrast, the activation of PVAT beiging attenuates these pathological changes. Mechanistically, neuregulin 4 (NRG4) secreted from beige PVAT promotes the alternative activation of macrophages, thereby facilitating the resolution of vascular inflammation [47]. Several experimental interventions support the therapeutic potential of PVAT browning. Kip et al. demonstrated that preoperative methionine restriction (MetR) enhances browning in thoracic and venous PVAT, which in turn improves vein graft remodeling and reduces postoperative pro-inflammatory responses [88]. Similarly, renal denervation in spontaneously hypertensive rats induces PVAT browning, increases adiponectin secretion, reduces reactive oxygen species (ROS) production, and attenuates hypertensive vascular remodeling [89]. Pharmacological agents such as luseogliflozin, a sodium-glucose cotransporter 2 (SGLT2) inhibitor, attenuate neointimal hyperplasia after wire injury in HFD–fed mice partly by suppressing PVAT whitening through inhibition of PDGF-B–expressing macrophage infiltration [90].

In summary, vascular remodeling, a pivotal pathological process in TAA, is profoundly influenced by the functional state of PVAT. Obesity-induced PVAT dysfunction exacerbates adverse remodeling by secreting paracrine factors that promote VSMC phenotypic switch and inflammation. Conversely, PVAT browning enhances metabolic and anti-inflammatory responses, exerting protective effects against pathological remodeling. This dual role of PVAT highlights its critical contribution to TAA pathogenesis. Therefore, therapeutic strategies aimed at promoting PVAT browning hold significant promise for mitigating vascular remodeling and ultimately preventing or attenuating TAA progression.

## PVAT Browning and Adventitia Remodeling

Adventitia has been increasingly recognized as a master regulator of arterial wall physiology and disease. Functioning as an information-processing center, it coordinates essential functions for vascular homeostasis and repair through complex communication with the immune and nervous systems, as well as with locally derived adipokines and microvascular cells [91–93]. Under pathological conditions, the adventitia becomes a critical site for inflammation, ROS production, and immune cell activation [94, 95]. It also undergoes fibrotic changes, which ultimately impair its capacity to compensate for medial weakening, thereby increasing the risk of aortic dilatation and rupture. Despite this emerging understanding, the contribution of adventitial pathology to TAA development has received relatively limited attention.

In large arteries, the outer adventitia merges with adjacent PVAT, and the two compartments are functionally linked through vasa vasorum microvessels, sympathetic and sensory nerves, and ECM components that bridge the interface [93, 96, 97]. This close anatomical and functional relationship enables dynamic crosstalk between PVAT and the adventitia, particularly during vascular disease. Dysfunctional PVAT exacerbates adventitial remodeling, a process further amplified in obesity, by promoting adventitial fibroblast proliferation and differentiation, notably via activation of the NLRP3/IL-1 signaling pathway [98]. PVAT dysfunction also leads to increased secretion of angiotensin II (Ang II), which stimulates adventitial fibroblast proliferation and collagen synthesis [99, 100]. Interestingly, accumulating findings suggest that the outside-in paracrine signaling originating from dysfunctional PVAT may be even more influential than previously appreciated. For instance, in hyperlipidemic, atherosclerosis-prone ApoE-deficient mice, inflammatory cell accumulation predominantly occurs in the adventitia rather than the intima, and in atherosclerotic human aorta, inflammatory cells were observed to be densely clustered in PVAT at the adventitial margin [101, 102]. Our previous study further demonstrated that mitoNEET-induced PVAT browning improved adventitial fibrosis and PVAT inflammation which prevented HFD-induced arterial stiffness [103]. These findings underscore the therapeutic potential of targeting PVAT browning to improve adventitial remodeling, though further studies are needed to clarify the underlying mechanisms.

### PVAT Phenotypic Shift and TAA

Genetic variants play a pivotal role in the pathogenesis of TAA, primarily through mutations affecting VSMCs. Accumulating studies indicate that perivascular adipocytes share progenitors with VSMCs [45, 96, 104]. However, it remains unclear whether—and how—mutations within PVAT contribute to TAA development. Recent investigations have begun to illuminate the active participation of PVAT progenitor cells in vascular pathology: these progenitors possess the capacity to migrate to sites of vascular injury, where they may differentiate into vascular cell lineages and influence disease progression.

Single-cell RNA sequencing (scRNA) has revealed two distinct subpopulations among PVAT-derived mesenchymal stem cells (PV-ADSCs), one of which features signaling pathways crucial for smooth muscle differentiation. In a mouse vein graft model, transplantation of red fluorescent protein (RFP)-labeled PV-ADSCs significantly enhanced neointima formation. Notably, RFP signals were detected within the neointima, and RFP-positive cells expressed the VSMC marker ACTA2 (α-smooth muscle actin). In vitro experiments further demonstrated that TGF-β1 induces PV-ADSC differentiation toward the SMC lineage. Collectively, these data indicate that PVAT-derived progenitors contribute to neointimal development through smooth muscle differentiation [105]. Supporting this, scRNA data from Xie et al. identified a Clec11a + subpopulation potentially involved in regulating PV-ADSC differentiation toward a SMC phenotype. Consistently, other studies have shown that Clec11a⁺ cells expressing mesenchymal stem cell (MSC) markers lack adipogenic potential, reinforcing their role in non-adipocyte lineage commitment [96, 106].

Pan et al. utilized scRNA to characterize young and aged PVAT-derived stromal cells (PVASCs) and found decreased endothelial and brown adipogenic differentiation capacities during aging. To investigate the pathological roles of young and aged PVASCs in vascular remodeling, the cells were embedded in Matrigel and implanted into the perivascular area of carotid arteries following ligation injury. Compared to the young PVASCs group, the implantation of aged PVASCs significantly promoted neointimal hyperplasia. Immunostaining revealed that young PVASCs differentiated into endothelial cells (CD31⁺) and myofibroblasts (αSMA⁺) within the neointima, and into brown adipocytes in the perivascular region. In contrast, aged PVASCs showed increased αSMA and decreased CD31 staining in the neointima and failed to differentiate into brown adipocytes. Notably, overexpression of PGC1α, a transcriptional coactivator that activates thermogenesis, restored the brown adipogenic potential of aged PVASCs and mitigated vascular remodeling [65].

SM22α^+^ cells accumulated in PVAT of Ang II-treated aged mice and patients with aortic aneurysms. ScRNA revealed that aging disrupts the differentiation potential of SM22-lineage PVASCs and leads to reduced PGC-1α expression. Using AAA mouse models with SM22α-driven PGC-1α deletion, the authors observed increased differentiation of PVASCs into myofibroblasts, which contributes to PVAT fibrosis. This indicates that PGC-1α is a key modulator that controls the balance between adipogenesis and myogenesis in PVAT, thereby inhibiting PVAT ECM accumulation and AAA development [54].

Collectively, during CVD or aging, subpopulations of PVAT progenitor cells undergo dynamic changes, showing a shift in differentiation potential from brown adipocytes toward vascular lineage cells. This transition contributes to pathological vascular remodeling. However, strategies that promote browning, such as overexpression of PGC1α, can restore the brown adipogenic potential of these cells, offering protective effects and helping to alleviate CVD. Despite these findings, there remains a limited understanding of the role that PVAT progenitors play in the development of TAA. Further studies are needed to elucidate this question, which may provide new perspectives on TAA pathophysiology and potential therapeutic targets, especially for genetically driven forms of TAA.

## Protective Effects of PVAT Browning in TAA

Transplantation of brown-like thoracic-PVAT onto the abdominal aorta significantly attenuates calcium phosphate–induced aneurysm formation compared to transplantation with abdominal-PVAT [64], underscoring the superior protective properties of browning PVAT. Correspondingly, TAA are less prevalent and progress more slowly than AAA [2, 107], suggesting that the BAT-like characteristics of thoracic PVAT may confer greater resistance to aneurysm development. However, the causal relationship between PVAT dysfunction and TAA pathogenesis, as well as the underlying mechanisms, remains to be elucidated. To address this gap, we analyzed aortic samples with adherent PVAT from TAA patients undergoing surgical repair. We observed a marked reduction in browning characteristics in PVAT adjacent to aneurysmal lesions. Subsequently, we employed two mouse models: brown adipocyte-specific PPARγ knockout (PPARγ^BAKO^, resulting in impaired PVAT development) and brown adipocyte-specific Prdm16 knockout (Prdm16^BAKO^, resulting in whitening and dysfunctional PVAT). Both PPARγ^BAKO^ and Prdm16^BAKO^mice exhibited aggravated elastase-induced TAA formation, indicating that both PVAT deficiency and PVAT dysfunction contribute to TAA formation. Mechanistically, we identified decorin, a small extracellular matrix proteoglycan, as a transcriptional repressive target of PRDM16. Elevated decorin levels were detected in both dysfunctional PVAT and the plasma of TAA patients, suggesting that PVAT-derived decorin may act as a paracrine mediator of PVAT dysfunction–driven TAA development [60].

**Fig. 1:**
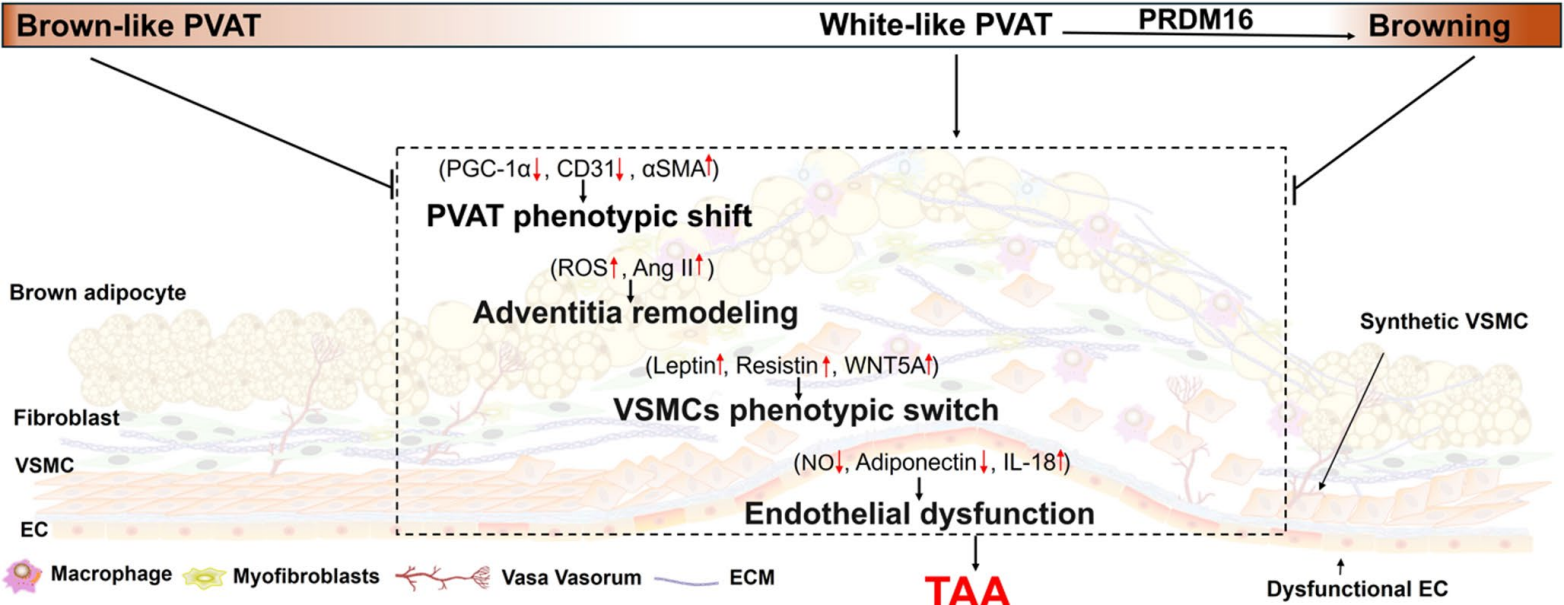
Proposed mechanism for the protective role of perivascular adipose tissue (PVAT) browning in thoracic aortic aneurysm (TAA). PVAT is highly plastic and metabolically active. In the early stages of TAA, PVAT becomes activated, enhancing its thermogenic capacity and exerting protective metabolic effects that slow disease progression. As TAA advances, PVAT gradually loses its brown adipose characteristics, marked by diminished thermogenic activity, adipocyte hypertrophy, and increased immune cell infiltration, leading to a transition toward a dysfunctional phenotype. PVAT dysfunction alters the expression of multiple signaling molecules that contribute to key pathological processes underlying TAA progression, including endothelial dysfunction, vascular smooth muscle cell (VSMC) phenotypic switching, adventitial remodeling, and further PVAT phenotypic shift. Dysfunctional, whitened PVAT exacerbates these events, whereas preservation of brown, metabolically active PVAT helps mitigate disease advancement. PRDM16-mediated PVAT browning holds promise as a potential pharmacological target for TAA therapy

Taken together, these findings strongly suggest that maintaining or promoting PVAT browning confers protection against TAA formation and progression (Fig. 1). Further investigation is warranted to delineate the precise mechanisms and to validate this therapeutic potential in clinical settings.

## PVAT Browning as Therapy for TAA

Currently, no therapies for CVD are designed to specifically target PVAT. Several important limitations continue to hinder the development of PVAT-directed therapeutic strategies: (1) Incomplete understanding of human PVAT pathophysiology across different forms of CVD. Much of the current knowledge is derived from animal models, which may not fully replicate PVAT function or behavior in humans. (2) Limited insight into the crosstalk between PVAT and the various layers of the aortic wall, including the intima, media, and adventitia. This complex interaction remains poorly defined, particularly in disease contexts. (3) Lack of PVAT-specific markers, which increases the risk of off-target effects. Most therapeutic interventions affecting PVAT may also act on other tissues or organs, leading to potential systemic or metabolic side effects. (4) Insufficient tools for non-invasive monitoring of PVAT status. Reliable imaging techniques and circulating biomarkers to assess PVAT phenotype and function in clinical settings are still lacking. Despite these challenges, several medications commonly used in cardiovascular and metabolic disease management, including GLP-1 receptor agonists [108], RAAS inhibitors [109], SGLT2 inhibitors [90], β3-adrenergic agonists [110], PPARγ agonists [111] and statins [112], can modulate PVAT function directly or indirectly, thereby helping to restore its vascular protective properties.

Among emerging molecular targets, PRDM16 has gained attention for its protective and regulatory roles in cardiovascular tissues and beyond [47, 113–115]. PRDM16, a transcriptional regulator highly expressed in BAT, is well recognized for its strong ability to induce the browning of adipose depots and to activate thermogenic and anti-inflammatory gene programs. In the context of PVAT, PRDM16-mediated browning holds strong therapeutic potential for cardiovascular diseases.

Beyond adipose tissue, recent studies have shown that PRDM16 plays protective roles in various pathological contexts across multiple organs and tissues. In the mammalian heart, PRDM16 is present in ventricular, but not atrial, cardiomyocytes [116, 117]. Transgenic and knockout animal studies have revealed that PRDM16 is essential for cardiovascular development and for maintaining cardiovascular function both under physiological and pathological conditions [113, 118]. In the kidney, PRDM16 overexpression or pharmacological induction improves renal dysfunction and fibrosis in diabetic mice [119], and protects against sepsis-associated acute kidney injury by suppressing ferroptosis [120]. In adult muscle pathology, overexpression of PRDM16 significantly improves muscle function and reduces fatty infiltration, a key marker of muscle degeneration and poor healing [121].

Within the vasculature, PRDM16 is predominantly expressed in arterial ECs and VSMCs of the aortic tree, while being absent in venous vascular cells. Endothelial cell–specific Prdm16 knockout (EC-Prdm16 KO) mice exhibit impaired arterial blood flow recovery due to endothelial dysfunction [122]. Smooth muscle–specific Prdm16 deletion leads to maladaptive SMC phenotypic switching, ECM remodeling, and inflammation, resulting in AAA formation [115] and the development of atherosclerosis [123]. Additionally, studies demonstrate that PRDM16 deficiency in VSMCs (Prdm16SMKO) disrupts the circadian variation of blood pressure in mice [114]. Mesenteric artery rings isolated from Prdm16SMKO mice show a diminished contractile response to phenylephrine, reflecting impaired adrenergic signaling and altered circadian regulation of clock genes [114]. Of particular relevance to PVAT, loss of PRDM16 induces a shift toward a white, pro-inflammatory phenotype (PVAT whitening), which fosters neointima formation and promotes TAA development [60].

Collectively, these findings highlight PRDM16 as a central regulator of tissue homeostasis and integrity across multiple organ systems. Its broad protective roles suggest that systemic augmentation of PRDM16 activity to promote PVAT browning may also confer benefits to other organs, with a lower likelihood of adverse side effects. Mechanistically, PRDM16-driven PVAT browning enhances its anti-inflammatory and metabolic functions, which exerts beneficial paracrine effects on the adjacent aortic wall. This not only improves EC function but also preserves the contractile phenotype and overall health of VSMCs, thereby mitigating pathological vascular remodeling. Consequently, therapeutic modulation of PRDM16 holds significant potential to attenuate the progression of atherosclerosis, hypertension, and TAA, highlighting its value as a novel target in vascular disease management.

## Conclusion

TAA remains a life-threatening condition for which surgical intervention as the main treatment, highlighting an urgent need for effective pharmacological strategies. Given its anatomical proximity and potent paracrine influence on the aortic wall, PVAT represents a compelling and underexplored therapeutic target. Its marked phenotypic plasticity, shifting between protective brown/beige states and dysfunctional white phenotypes, critically shapes vascular inflammation, remodeling, and aneurysm progression. Although human PVAT data remain limited, robust animal studies consistently demonstrate that promoting PVAT browning attenuates key pathological drivers of TAA, including endothelial dysfunction, VSMC phenotypic transition, adventitial remodeling, and PVAT phenotypic shift. Targeting PVAT browning pathways, such as PRDM16, or leveraging pharmacological browning agents therefore holds significant promise for TAA intervention. Advancing this therapeutic direction will require deeper mechanistic insight into human PVAT biology, improved understanding of PVAT–aorta interaction, and the development of PVAT-specific imaging tools and biomarkers. Addressing these gaps will be essential to translate PVAT-focused strategies into clinically meaningful treatments for TAA.

##  Key References

- Wang Z, Lu H, Garcia-Barrio M, Guo Y, Zhang J, Chen YE, et al. RNA sequencing reveals perivascular adipose tissue plasticity in response to angiotensin II. Pharmacol Res. 2022;178:106183.
  - This is the first study to systematically investigate the regional heterogeneity of PVAT at different location of aorta using bulk RNA-seq and to explore the distinct regional responsiveness of PVAT to Ang II.
- Adachi Y, Ueda K, Nomura S, Ito K, Katoh M, Katagiri M, et al. Beiging of perivascular adipose tissue regulates its inflammation and vascular remodeling. Nat Commun. 2022;13(1):5117.
  - This study shows that vascular injury induces the beiging of PVAT, while inhibition of PVAT beiging by genetically silencing PRDM16 exacerbates inflammation and vascular remodeling following injury, demonstrating the protective role of PVAT beiging in vascular inflammation and remodeling.
- Wang Z, Mu W, Xu R, Zhong J, Xiong W, Zhao X, et al. Perivascular adipose tissue dysfunction contributes to thoracic aortic aneurysm development. Cardiovasc Diabetol. 2025;24(1):223.
  - This is the first study to demonstrate that the development and maintenance of brown-like characteristics in PVAT are necessary to protect against TAA formation, providing a promising therapeutic strategy for preventing TAA progression by inducing PVAT browning.
- Pan X, Zhang R, Lu B, Chen S, Chen H, Li M, et al. SM22α-Lineage Perivascular Stromal Cells Contribute to Abdominal Aortic Aneurysm. Circ Res. 2025;137(1):4-22.
  - This study focuses on the mechanisms underlying PVAT plasticity in aneurysm pathogenesis. It demonstrates that PGC-1α deficiency in SM22α-lineage PVSCs disrupts the balance between adipogenic and myofibrogenic differentiation by regulating YAP signaling, ultimately promoting AAA development.

## Data Availability

No datasets were generated or analysed during the current study.
